# Clinical Methods

**Published:** 1906-02

**Authors:** 


CLINICAL METHODS. By Robert Hutchison, M.D., F. R., C. P., Assistant Physician to London Hospital, etc., and Harry Rainy, M. A., F. R. C. P., Examiner in Medicine and Clinical Medicine, St. Andrews University, etc. With upwards of 150 illustrations and 9 colored plates. Ninth edition. Cloth. 12mo. Pp. 636. Price $2.50 net. W. T. Keener & Co., Chicago.

"How shall I investigate this case?" According to the preface of the original edition, this work was intended to provide an answer to that question. The demand for seventeen thousand copies during the last eight years is evidence that the above question was correctly as well as ably answered. We commend the book to all who desire a brief, clear treatise on the methods of medical diagnosis.

